# On the External Use of the Extract of Belladonna

**Published:** 1826-11

**Authors:** Thomas Wm. Chevalier

**Affiliations:** Consulting Surgeon to the Royal Union Association, and acting Surgeon to the Westminster General Dispensary.


### EXTERNAL APPLICATION OF BELLADONNA.

*On the External Use of the Extract of Belladonna.* By Thomas Wm. Chevalier, Consulting Surgeon to the Royal Union Association, and acting Surgeon to the Westminster General Dispensary.

It follows, from the nature of all *power,* and particularly from that of the *vis insita musculorum,* that an excess of vascular action cannot take place without either excessive excitement, or excessive sensibility to ordinary excitement.

To remove irritation has, therefore, always been considered one chief object of medical and surgical treatment; and, indeed, if it could be accomplished under all circumstances, and to an unlimited extent, every kind of morbid inflammation would be curable.

There are many circumstances, however, which limit both the physician and the surgeon, in their attempts to diminish extraordinary *vascular excitement;* for there are many cases in which the immediate cause of such excitement cannot be removed; and there are some in which it cannot even be diminished; so that in these there remains only the hope of allaying the *sensibility* of the part affected. And, although we have various means of allaying the sensibility or irritability of particular organs, as well as of the whole body, yet, even in this intention, our power is greatly limited; for most sedatives impair the natural functions in nearly the same ratio in which they alleviate disease, and we have scarcely any very powerful sedative, at present in general use, that can be so applied as to alleviate local disease, without affecting also other parts which are in health, or even the constitution of the patient, both unnecessarily and injuriously.

The Extract of Belladonna is the strongest sedative of which the College of Physicians authorises the employment. It is a remedy of which the effect is most uncertain, when it is internally administered; but, when applied in the form of an ointment or of a plaster, it has been found by the author to exercise a most decided and most beneficial effect upon the parts beneath it, without at all affecting the constitution of the patient, or any other organ than that on which it is intended to operate.

In the first of the following cases, the author was enabled, by means of this extract, successfully to employ remedies, which otherwise it had been impossible to use; in two others a complete cure was obtained of limbs condemned to amputation; and by the rest it appears established, that inflammation, of the most obstinate and destructive kind, may frequently be subdued by a judicious application of the same external sedative.

A gentleman, of unusually firm mind, applied to me, in July last, under the following circumstances:—He had contracted a gonorrhœa five years before, which was relieved by the use of injections, but left a gleet that had never since been entirely cured. Of late he had suffered severely from pain in the region of the left kidney, and he informed me that he had been under treatment for a stricture; his former medical attendant (whose name I forbear to mention,) having passed bougies of a size not exceeding No. 7, and directed him to wear them in his urethra, frequently during a walk of two miles. On the 15th, I introduced the largest bougie he would allow me to use, (viz. No. 7,) and discovered a mechanical obstruction in the membranous part of the urethra, anterior to which (viz. at about two inches from the external orifice,) a portion of the canal, about half an inch in extent, was so intensely irritable as not only to resist for some minutes the passage of the instrument, but also to render its introduction so intolerably painful, that the perspiration broke out in beads upon the patient's forehead: in short, he manifestly suffered more than any one would or could submit to frequently. Under these circumstances, although I believe it had never been done before, I resolved to anoint the bougie with the extract of belladonna, at first mixed with that of opium, and afterwards pure, being merely moistened with water; and I sometimes intentionally left a portion (I should imagine from one grain to three) of the pure extract of belladonna in the irritable part of the urethra. After the bougie, thus prepared, had pressed upon the irritable spot for about two minutes, the intense pain which my patient at first endured was mitigated, so much that I continued to pass larger bougies in succession, until at length No. 14 was introduced without any difficulty. The pain in the region of the kidney was relieved, and finally cured, under the application of a plaster composed of one-third Extractum Belladonnæ and two-thirds Ceratum Saponis, with an occasional blister. The gleet was got rid of by means of astringent injections, together with the nocturnal use of one which I have found very useful in other cases of the kind, and in several cases of gonorrhœa, and which is composed of an infusion of cubeba, in the proportion of an ounce to a pint, with a scruple of the extract of belladonna.

I may here add, that I have used a solution of the Extractum Belladonnæ, combined with other ingredients, and especially the vegetable astringents, in many other cases of irritable urethra and of morbid tenderness in the vagina, with similar success.

To scrofulous glands I have applied the belladonna ointment, (composed of equal parts of the extract and of some other ointment,) sometimes with striking advantage: in these, as in other cases, I employ it where *the treatment of allaying irritation, by means of decreasing the sensibility of parts*, is especially indicated as a means of cure, or as the only means of relief.

In the case of Charles Bloquet, from an abscess in whose neck I let out more than half a pint of fetid pus with some sloughy shreds, I cannot but believe that I obtained an uniformly progressive and complete recovery, principally by the use of the belladonna ointment, as often as the inflammation threatened to become active. The extensive induration over all one side of this man's throat was, however, dispersed by the use of the Unguentum Hydriodatis Potassæ; the belladonna being employed beneath a poultice as often as a return of suppurative inflammation was threatened.

In many cases of inflamed periosteum, in cases of venereal node, and also more especially in cases of scrofulous disease of the bones and joints, and those affections of these parts which arise from the abuse of mercury, the extract of belladonna, as a soothing application, is exceedingly valuable. As every other remedy, it must, however, be employed judiciously and upon principle, to exhibit its power. Let us, therefore, consider in how many such cases constitutional remedies would effect a cure, and that a perfect one, could we but *delay* the progress of the local morbid action: and be it observed, that the extract of belladonna, although a very powerful sedative, is far from being the only one, or even the best, in many cases, that might be employed, upon similar principles, with inestimable benefit. In one case, where a tumor, as large as half a small orange, had formed upon the back of a labouring man's right hand, so that the hand was condemned to be amputated, and as I myself believed must have been so, the swelling dispersed in less than ten weeks, under the belladonna plaster, in the first instance, and afterwards the pure extract.

Again, Matthew Hill, a boy of fourteen years old, of scrofulous constitution and delicate form, came to me at the Dispensary in August last. I found him suffering, on the 25th, from severe pain in the left knee-joint, which had been bent during the last five years, and imperfectly anchylosed, at almost a right angle; the condyles were half as large again as those of the other knee; the capsular ligament was distended with fluid, and (as the severity of the pyrexial symptoms indicated) with pus; and it was pointing on the inside, where the skin was prominent and inflamed. By the application of leeches, a poppy fomentation, and narcotic poultice, with the free administration internally of equal parts of laudanum and antimonial wine, the severity of the symptoms was subdued. Amputation, however, was insisted upon by myself, and upon the opinion of Mr. Copland Hutchison; but the boy refused to submit to it: I therefore commenced the use of belladonna plasters, and the joint from that time continued to diminish in size, and to become less sensible; while the boy's general health was much improved by the use of tonics and generous diet. In October, I commenced the use of splints over plasters of the pure extract of belladonna, covering the whole knee; and, by changing the splints for new ones made purposely for him, and adapted to his limb, so that it was always retained at its proper degree of distention in perfect rest, I was enabled to bring the leg and thigh to an angle of 150°, the knee-joint being little larger than the other; and I have no doubt that I should have completely straightened it, but that the boy was of too volatile a disposition to care about his crooked limb, when set in comparison with close confinement; so that he left off coming to the dispensary. He took at different times cinchona, steel wine, and sulphate of zinc.

Upon the above principle I have straightened several limbs, but much perseverance is required, both on the part of the surgeon and his patient; and also considerable expence must be incurred for splints.

In cutaneous affections, the belladonna is frequently efficacious. In several cases of herpes, and in some of very many months' continuance, the disease has been cured in a week or two with the ointment. In several cases of a cutaneous affection, like herpes, which is common in the face of young children, and in one of this kind which had long baffled every other mode of treatment, the use of the belladonna ointment, beneath gold-beater's skin, has cured the disease, though of some years standing, in as many weeks. I should here observe, however, that this ointment has, in one or two cases of herpes, rather done harm than good, where the vessels of the part were previously much debilitated.

In some cases of cutaneous ulcerations, with much induration, from scrofula, and in other cases of extremely irritable ulcers, wherein the ulcerative character is most strongly marked, I have employed the belladonna with advantage, either as applied over the dressings to the surrounding skin, or immediately to the sore. This is the more remarkable, inasmuch as the belladonna plaster, if left on too long after it begins to annoy the patient, is capable of producing small sores of a purely ulcerating character, though to be readily healed by mild cerates.

Incipient abscesses have been resolved, and in one case, most decidedly, I was enabled to prevent an extravasation of blood beneath the fascia from suppurating, by the application of belladonna.

In the case of Mary Dryden, who applied to me at the dispensary for an obstinate cutaneous inflammation of three years' standing, which had left a cicatrix over the whole of one shoulder and breast, and had traversed the throat, being now seated on the nose and eyelids, in the form of an angry scabbing superficial ulceration, not very different from noli-me-tangere, a rapid cure was obtained by the use of sarsaparilla and the belladonna ointment; namely, in less than five weeks.

I have used the belladonna ointment in cases of erysipelatous inflammations: in these it is not equal in efficacy to spirituous applications, and far inferior to the Eau de Cologne. The case in which I derived most advantage from its employment, was one of œdematous ankles, attacked with erysipelas from scarification, and affected with intense pain and tenderness: in this case it accomplished complete relief in three or four days.

The servant of a friend of mine had been suffering, in the year 1824, during more than nine months, from all the symptoms of ulceration in the kidney, with retention of urine. She was last year attacked, more severely than ever before, with the same disease, and yet she was completely relieved from every symptom in four or five days, by large quantities of opium, castor, and valerian, internally administered, while a plaster of belladonna and soap cerate was applied to the region of the bladder. In other cases of a similar nature I have seen this remedy most decidedly efficacious.

I have used the belladonna ointment to cancers in a state of ulceration, with the most decided advantage; but in these cases it has been only of the strength of from one-sixth to a fourth part extract. An ointment composed of one-sixth flowers of digitalis and five parts fresh butter, (prepared by boiling until the flowers are crisp, and straining,) is also a very efficacious means of diminishing the activity of cancerous action. This ointment was much used by my late father in such cases, and with extraordinary success.

In inflammatory and spasmodic affections of the thoracic viscera, I have employed belladonna plasters with singular relief, applying them to the seat of pain or between the mammæ, from the size of a duodecimo to that of a large octavo page. Some degree of caution, however, is necessary; for in those who have been rendered irritable by the long continuance of internal disease, whose pulse is weak and varying as to its frequency, the hand unsteady, and the whole frame debilitated beyond what might be expected from the first appearance of the patient, the belladonna plaster will occasionally, though very rarely, affect the pupil; the sight becoming dimmed, and the head disordered. I know of one case in which a large belladonna plaster, applied to the loins of such a subject, produced a degree of paralysis of the levator palpebræ superioris, which continued many weeks afterwards, and only got well at last under repeated blisters applied to the temple. Where the debility of the patient, although considerable, does not exceed what the general appearance of her frame would indicate, I have not seen these ill effects, although I have extensively employed the remedy in such persons.

In cases of tooth-ache, of abscess with severe pain, partial acute rheumatism, &c. &c. I had long been accustomed to use not only pure laudanum as an embrocation, but also to rub the neighbouring skin with a solution of Extractum Opii, sometimes of the consistence of honey; and, excepting in children, I never saw any inconvenience arise from this treatment. The late Mr. Griffiths, surgeon to St. George's Hospital, was in the habit of applying the black drop to the most extensive burns: I have applied it, by his order, to more than half the surface of a child's body, and I never knew any case in which it did the slightest harm. I have heard, indeed, of a child's dying suddenly under the application: and who has not known children severely burned die suddenly under the simplest treatment.

Encouraged by these facts, and accounting the surest remedy, if demonstrated to be safe, as the first to be used in all cases, I caused my patients with acute rheumatism in a single limb, for example, or in the scalp, chest, &c. to rub the part affected with an ointment composed of from an eighth to a fourth of Extractum Belladonnæ, with a few drops of Oleum Lavendulæ Anglicanum, and the rest lard; and by this means the pain is almost certainly relieved, and the disease subdued. In one case, as often as the ointment was used, the rheumatic inflammation ceased, reappearing in another limb; recurring in this way six or eight times. I am, however, accustomed to direct that the ointment should be rubbed upon the part only until the pain *begins* to abate, and left upon it afterwards only while the pain continues to be present.

I should think there cannot be less than between two and three hundred persons, to whom I have applied the belladonna plaster or the ointment, in my private practice and at the Westminster Dispensary, (where I have been appointed to act for one of the surgeons, Mr. A. C. Hutchison, during two years,) and the cases are very few in which it has failed to do good; none in which it appears to have done harm. I am, however, far from presuming that it is incapable of producing the most serious mischief, if imprudently employed, or if it should not be removed as soon as ever the sight is the least affected by it. The facts contained in this paper are intended only to prove that it is a powerful and safe mean of allaying the sensibility of parts; and that, with proper care and judgment, it accomplishes this important end without at all affecting distant parts,—still less, the constitution generally.

A good deal of care is requisite in preparing both the ointment and the plaster, or the one will not be thoroughly mixed, and the other will not adhere.

In concluding this paper, I feel myself called upon to acknowledge my obligations to my friend Mr. Powel Blackett, of Park-street, Grosvenor-square, to whose paper in this valuable Journal I am indebted for the first inducement I received to make an extensive trial of the belladonna, as well as for the proportions of the extract in the ointment and plaster.

20, *South Audley-street; July* 12*th*, 1826.

